# Altered early-life gut microbiota in offspring of pregnancies complicated by CHD-associated pulmonary hypertension

**DOI:** 10.3389/frmbi.2026.1785707

**Published:** 2026-05-29

**Authors:** Yiyang Han, Haofeng Zhang, Jun Zhang

**Affiliations:** 1 Department of Cardiac Surgery, Beijing Anzhen Hospital, Capital Medical University, Beijing, China; 2 Beijing Institute of Heart, Lung and Blood Vessel Diseases, Beijing, China

**Keywords:** 16S rRNA, congenital heart disease, early-life neonatal gut microbiota, pregnancy, pulmonary arterial hypertension

## Abstract

**Background:**

Pulmonary arterial hypertension is a progressive disease involving the pulmonary vasculature and is defined as a mean pulmonary arterial pressure (mPAP) >20 mmHg at rest. Pulmonary arterial hypertension during pregnancy is associated with increased maternal mortality and adverse fetal outcomes. The present study aimed to investigate differences in the initial meconium microbiota between neonates born to mothers with congenital heart disease-associated pulmonary arterial hypertension (CHD-PAH) and those born to mothers with congenital heart disease (CHD) alone, thereby elucidating the potential influence of pulmonary arterial hypertension on the establishment of the early-life gut microbiome.

**Methods:**

We collected first-pass meconium samples from neonates in the pulmonary hypertension group (PH group, *n* = 23) and the control group without pulmonary hypertension (NC group, *n* = 17) and characterized microbial profiles using 16S rRNA sequencing.

**Results:**

The PH group showed lower alpha diversity, with reduced Shannon and observed features indices (both *P* < 0.05), whereas Bray–Curtis beta diversity showed substantial overlap between groups. At the phylum level, the overall gut microbial structure was broadly comparable between the PH and NC groups, with no statistically significant differences in the relative abundance of dominant taxa. At the genus level, the mean relative abundance of *Streptococcus* was significantly lower in the PH group than in the NC group (0.20% vs. 2.09%, *P* = 0.0072). Predicted functional profiling suggested potential differences in dominant metabolic pathways between groups, including enrichment of ubiquinone biosynthesis and aromatic amino acid/chorismate biosynthesis pathways in the PH group.

**Conclusion:**

Collectively, these findings extend current evidence on PAH-related alterations in early-life microbial ecosystems and provide a plausible microbiome-based basis for investigating the biological mechanisms underlying adverse maternal–fetal outcomes associated with pulmonary arterial hypertension.

## 1 Introduction

Pulmonary arterial hypertension is a progressive disease involving the pulmonary vasculature and is defined as a mean pulmonary arterial pressure (mPAP) >20 mmHg at rest. Pulmonary arterial hypertension during pregnancy is associated with an increased risk of maternal mortality and adverse fetal outcomes. Notably, reports from the 1990s documented a maternal mortality rate exceeding 50% among pregnant women with pulmonary arterial hypertension (Benza et al., 2012; McGoon et al., 2013). In recent years, with improvements in diagnostic accuracy and therapeutic strategies, maternal mortality associated with pulmonary arterial hypertension (PAH) in pregnancy has declined. The most common form of PAH is congenital heart disease (CHD)-associated PAH; the maternal mortality among pregnant women with CHD-related moderate-to-severe PAH has been reported to be 5.7% (Chin and Santiago-Munoz, 2023), whereas patients with CHD-related mild PAH appear to have a more favorable prognosis, with a maternal mortality of 0.1% (Chin and Santiago-Munoz, 2023). Nevertheless, despite this encouraging downward trend, PAH remains classified by the World Health Organization as a contraindication to pregnancy, largely because of the high burden of cardiovascular complications and the persistently elevated risk of adverse fetal outcomes (Rudienė et al., 2022). In studies investigating the fetal impact of maternal disorders during pregnancy, conditions such as gestational diabetes mellitus and preeclampsia have been shown to influence the composition of the neonatal gut microbiota (Chen et al., 2021; Chen et al., 2025). Genetic factors, medication exposure during pregnancy, and mode of delivery have also been implicated as important determinants of the early-life neonatal gut microbiome, collectively shaping early microbial colonization patterns and potentially modulating downstream immune and metabolic development (Mitchell et al., 2020; Yang et al., 2020; Cm et al., 2021).

Against this background, neonatal meconium, which is formed *in utero* and is considered to represent the earliest microbial community, has been widely used as a “window” to investigate fetal microbial colonization and its potential associations with early-life health (Turunen et al., 2024). Pregnant women with congenital heart disease-associated pulmonary arterial hypertension often experience hypoxemia and altered cardiocirculatory and metabolic states, which place the fetus in a chronically hypoxic intrauterine environment, and animal studies have demonstrated that prenatal hypoxia can modify gut microbial colonization in the offspring (Sun et al., 2021). Therefore, by comparing the meconium microbiota of neonates born to mothers with CHD-PAH with that of neonates born to mothers with CHD alone, we sought to determine whether pulmonary arterial hypertension exerts a specific and distinguishable influence on early-life neonatal gut microbial colonization.

In this study, we enrolled neonatal meconium samples from pregnancies complicated by congenital heart disease-associated pulmonary arterial hypertension and from those with congenital heart disease alone, thereby establishing a pulmonary hypertension group (PH group, *n* = 23) and a control group (NC group, *n* = 17), and characterized the microbial profiles using 16S rRNA sequencing. Based on rigorous quality control and standardized analytic workflows, we systematically compared intergroup similarities and differences in microbial composition, distribution patterns, and predicted metabolic functions, to delineate clues that the maternal pathological milieu may influence fetal early gut colonization through microbiome-related pathways and of providing biological evidence to better elucidate the mechanisms underlying PAH-associated maternal–fetal outcomes.

## 2 Materials and methods

### 2.1 Study design

This study was approved by the Institutional Ethics Committee of Beijing Anzhen Hospital, Capital Medical University, under approval number KS2024044. Pregnant women with congenital heart disease who were admitted to our hospital were consecutively enrolled, and maternal and fetal characteristics were obtained from the electronic medical record system. The inclusion criteria were as follows: First, mothers had congenital heart disease, with or without pulmonary arterial hypertension, that was diagnosed before or during pregnancy, and they delivered a singleton live birth at our institution. Second, maternal data on height, weight, and ethnicity, as well as information required to accurately determine gestational age, were available from the medical records. Third, maternal blood pressure and blood glucose remained within the normal range throughout pregnancy. Blood pressure was controlled at <140/90 mmHg during pregnancy. On the 75-g oral glucose tolerance test performed between 24 and 28 weeks of gestation, fasting blood glucose and blood glucose levels at 1 and 2 h after glucose ingestion were all below 5.1, 10.0, and 8.5 mmol/L, respectively.

The exclusion criteria included twin or multiple gestation and the presence of other categories of heart disease identified before or during pregnancy, including cardiomyopathy, valvular heart disease, aortic dissection, and coronary artery disease.

We divided the cohort into two groups according to whether maternal pulmonary arterial hypertension was present, and we defined a congenital heart disease cohort with pulmonary arterial hypertension as the PH group and a congenital heart disease cohort without pulmonary arterial hypertension as the NC group. Right heart catheterization is the diagnostic gold standard for pulmonary arterial hypertension, which is defined by a mean pulmonary arterial pressure greater than 20 mmHg. In clinical practice, echocardiography is more commonly used to estimate systolic pulmonary arterial pressure. In this study, mothers were assigned to the PH group when the highest estimated systolic pulmonary arterial pressure during pregnancy was at least 35 mmHg (Sun et al., 2018; Marshall et al., 2021), whereas the remaining mothers were assigned to the NC group. All mothers in this cohort had no history of diabetes, hypertension, intrahepatic cholestasis of pregnancy, smoking or alcohol consumption, autoimmune disease, or gastrointestinal surgery. Written informed consent was obtained from the legal guardians of all participants.

### 2.2 Sample collection

The first-pass meconium samples were collected within 24 h after delivery. Sampling was performed by trained personnel who followed a unified standard operating procedure. Approximately 2–5 g of fecal material was collected from a clean disposable diaper using sterile cotton swabs and transferred into sterile containers, after which the samples were transported to the laboratory in insulated containers maintained at 4°C and stored at −80°C until total DNA extraction for subsequent sequencing.

### 2.3 DNA extraction and sequencing

Genomic DNA was extracted using a fecal nucleic acid extraction kit from BeaverBio, catalog number 70411-100, according to the manufacturer’s instructions. After dilution to 5 ng/μL, PCR amplification was performed with 16S rRNA V3–V4 primers, in which the 341F primer sequence was CCTAYGGGRBGCASCAG and the 806R primer sequence was GGACTACNNGGGTATCTAAT, together with Taq HS Low DNA enzyme from TaKaRa, catalog number R090. Library construction and amplicon purification were carried out with Nextera XT Index primers from Illumina, catalog number FC-131-2001, and the amplified products underwent adaptor and index PCR using Tks Gflex DNA Polymerase from TaKaRa, catalog number R060. Quality control was performed with Qubit quantification and 2% agarose gel electrophoresis. Libraries that met the criteria for a concentration greater than 10 ng/μL and appropriate fragment size distribution were sequenced on the NovaSeq 6000 platform using the PE250 mode.

### 2.4 16S rRNA gene sequencing analysis

Paired-end sequencing data were first demultiplexed according to barcode information, after which reads were merged and filtered. Primer sequences were removed with cutadapt version 4.9 (Kechin et al., 2017), and overlapping reads were assembled into longer tags with FLASH, followed by sliding-window quality scanning of sequencing reads with fqtrim version 0.9.7 to remove low-quality sequences. The resulting data were processed with DADA2 implemented in QIIME2 version 2024.10.1 (Bolyen et al., 2019) for denoising and chimera removal to generate amplicon sequence variants (ASVs) and the corresponding ASV abundance table.

Alpha and beta diversity were assessed with the QIIME diversity module. Beta diversity was primarily evaluated by Bray–Curtis distance, and principal coordinate analysis was performed in R with the vegan package (Minchin, 1987), while group differences were assessed using permutational multivariate analysis of variance (PERMANOVA) implemented with the adonis function (Chapman and Underwood, 1999). Taxonomic assignment was conducted against the SILVA Ref NR 99 database (version 138.1) based on the ASV sequence file, and taxon abundances were summarized across samples according to the ASV abundance table. Key microbial biomarkers were identified using LEfSe implemented in the microeco package in R (Segata et al., 2011). Functional prediction was performed with the PICRUSt2 plugin in QIIME2 (Langille et al., 2013), which generated abundance tables for metabolic pathways, enzymes, and KOs, and the sweep function in base R was used to convert these tables into relative abundances for downstream functional profiling of pathway enrichment. Genus-level association analysis was performed using the 30 most abundant genera across all samples. Raw genus-level count data were transformed using a centered log-ratio (CLR) transformation after adding a pseudocount of 1 to account for the compositional nature of 16S amplicon data. Pairwise correlations between genera were then calculated, and *P*-values were adjusted for multiple comparisons using the Benjamini–Hochberg false discovery rate procedure.

## 3 Results

### 3.1 Participants and sequencing characteristics

First-pass meconium samples were collected within 24 h after birth from the PH group (*n* = 23) and the NC group (*n* = 17). The participants had similar dietary habits and lifestyles, and there were no statistically significant differences between the NC and PH groups in maternal age, pre-pregnancy BMI, gestational weight gain, premature rupture of membranes, mode of delivery, or neonatal Apgar scores (**Table 1**). Significant between-group differences were observed in gestational age at delivery as well as neonatal length and birth weight (*P* < 0.05).

**Table 1:** Baseline characteristics of mothers and neonates in the PH and NC groups included in this study.

Variable	NC	PH	*P*-value
*N*	17	23	<0.001
GA weeks	38.14 (38.00, 39.43)	38.00 (31.64, 38.57)	**0.030**
Weight gain (kg)	13.00 (8.00, 14.00)	10.00 (8.00, 12.00)	0.372
Age (years)	33.00 (29.00, 38.00)	31.00 (29.00, 35.50)	0.773
Apgar 1 min, *n* (%)			0.872
8	1 (4.35%)	1 (5.88%)	
9	4 (17.39%)	2 (11.76%)	
10	18 (78.26%)	14 (82.35%)	
Apgar 5 min, *n* (%)			0.638
8	1 (4.35%)	0 (0.00%)	
9	2 (8.70%)	1 (5.88%)	
10	20 (86.96%)	16 (94.12%)	
Apgar 10 min, *n* (%)			0.638
8	1 (4.35%)	0 (0.00%)	
9	2 (8.70%)	1 (5.88%)	
10	20 (86.96%)	16 (94.12%)	
Baby gender, *n* (%)			0.523
Male	7 (41.2%)	13 (56.5%)	
Female	10 (58.8%)	10 (43.5%)	
Baby height (cm)	50.00 (50.00, 50.00)	49.00 (42.00, 50.00)	**0.033**
Baby weight (g)	3,460.00 (3,120.00, 3,620.00)	2,690.00 (1,485.00, 3,185.00)	**0.004**
Delivery mode, *n* (%)			1.000
CS	15 (88.2%)	21 (91.3%)	
VD	2 (11.8%)	2 (8.7%)	
PROM, *n* (%)	2 (8.70%)	2 (11.76%)	1.000
SGA, *n* (%)	0 (0.00%)	1 (5.88%)	0.878

Bold *P*-values indicate statistical significance with *P* < 0.05.

GA weeks, gestational age in weeks; CS, cesarean section; VD, vaginal delivery; PROM, premature rupture of membranes; SGA, small for gestational age.

### 3.2 Diversity and structure of the early-life neonatal gut microbiota in neonates born to CHD-PAH mothers

**Figure 1:**
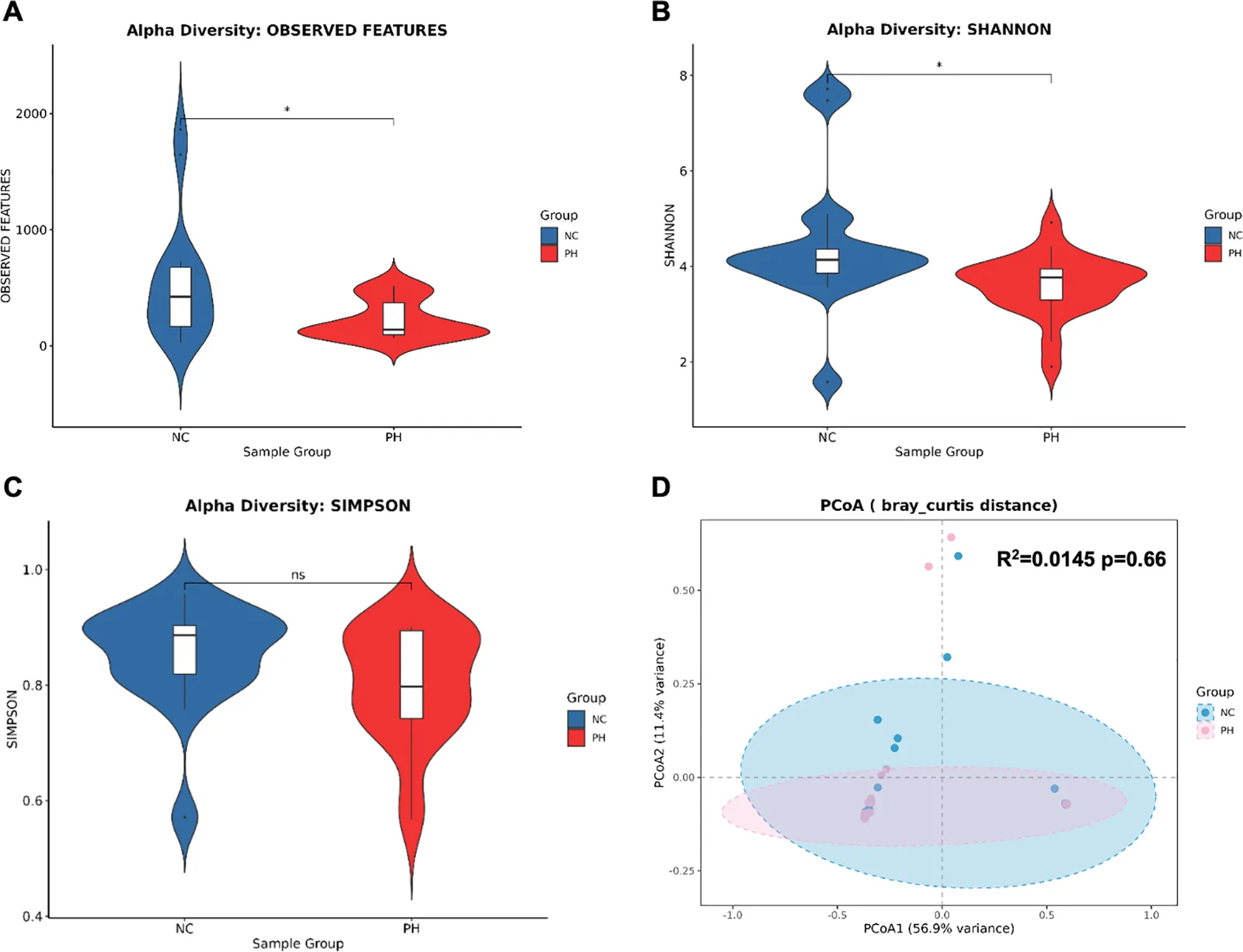
**(A–C)** Violin plots of a-diversity indices, including Observed species, Shannon, and Simpson. Between-group comparisons were performed using the t-test. *P < 0.05; ns, not significant. **(D)** Scatter plot showing β-diversity, which reflects differences in the taxonomic composition and community structure of the gut microbiome across groups. Samples are plotted along the first and second principal coordinates, and the proportion of variance explained by PCoA1 and PCoA2 is provided for each axis. Ellipses indicate the 95% confidence intervals for each group.

To investigate differences in the early-life neonatal gut microbiota between the PH and NC groups, we assessed the two groups using both α-diversity and β-diversity metrics. For α-diversity, Good’s coverage exceeded 99% in both groups. Compared with the NC group, the PH group showed significant differences in the Observed species (P = 0.0283) and Shannon (P = 0.040) indices (**Figures 1A, B**), whereas no between-group difference was observed in the Simpson index (P = 0.117) (**Figure 1C**). Principal coordinates analysis based on Bray–Curtis distances showed substantial overlap between the PH and NC groups overall, and PERMANOVA did not indicate a significant between-group difference (R^2^ = 0.0145, P = 0.66). Further dispersion analysis likewise did not support a significant between-group difference in within-group heterogeneity (mean distance to centroid: NC = 0.572, PH = 0.532; permutation test P = 0.201) (**Figure 1D**).

### 3.3 Microbial composition of the early-life neonatal gut microbiota in neonates born to CHD-PAH mothers

**Figure 2:**
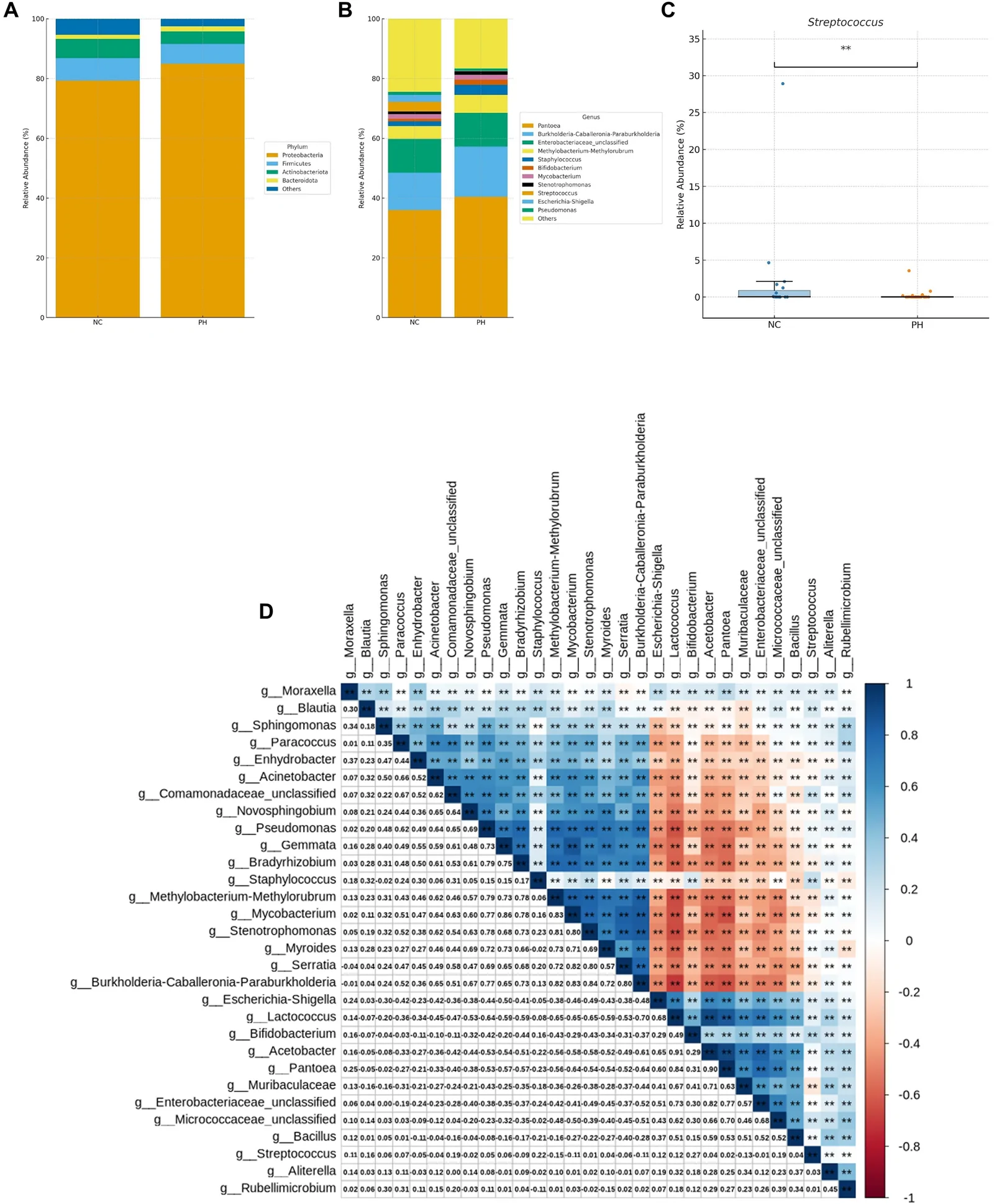
**(A, B)** Bar plots showing the composition of the gut microbiota at the phylum and genus levels. **(C)** Box plots showing the relative abundance of *Streptococcus* in the two groups. Comparisons between groups were performed using the rank-sum test. **(D)** Genus-level correlation analysis in which color indicates the strength of correlation, with darker colors representing stronger correlations. Negative values indicate negative correlations, whereas positive values indicate positive correlations. Statistical significance is denoted by asterisks, where * indicates *P* < 0.05 and ** indicates *P* < 0.01. The lower-left triangle displays the corresponding correlation coefficients.

By comparing the neonatal gut microbial composition between the NC group and the PH group, we sought to identify potential microbial features associated with PH. At the phylum level (**Figure 2A**), Proteobacteria (accounting for 79.30% and 84.96% of the total bacteria in the NC and PH groups, respectively) was the overwhelmingly dominant phylum in both groups. Firmicutes (7.54% and 6.64%), Actinobacteriota (6.41% and 4.16%), and Bacteroidota (1.42% and 1.67%) collectively constituted more than 90% of the microbial community in each group. No apparent differences were observed in the relative abundances of the dominant phyla between the two groups. Among low-abundance taxa, the mean relative abundance of environmental-associated microorganisms was lower in the PH group than in the NC group.

At the genus level (**Figure 2B**), the dominant taxa (mean relative abundance >1%) were as follows: *Pantoea* (accounting for 36.01% and 40.46% of the total bacteria in the NC and PH groups, respectively), *Burkholderia*–*Caballeronia*–*Paraburkholderia* (12.45% and 16.79%), Enterobacteriaceae_unclassified (11.33% and 11.27%), *Methylobacterium*–*Methylorubrum* (4.29% and 6.03%), *Staphylococcus* (1.61% and 3.35%), *Bifidobacterium* (0.84% and 1.72%), *Mycobacterium* (1.54% and 1.60%), *Stenotrophomonas* (0.91% and 1.18%), *Streptococcus* (3.21% and 0.13%), *Escherichia*–*Shigella* (2.35% and 0.06%), and *Pseudomonas* (1.04% and 0.82%). Enterobacteriaceae-related genera, *Staphylococcus*, *Bifidobacterium*, and *Mycobacterium* are the commonly observed dominant taxa in neonatal meconium, which is consistent with previous reports. Notably, the relative abundance of *Streptococcus* was significantly lower in the PH group than in the NC group (0.20% vs. 2.09%, *P* = 0.0072) (**Figure 2C**).

**Figure 3:**
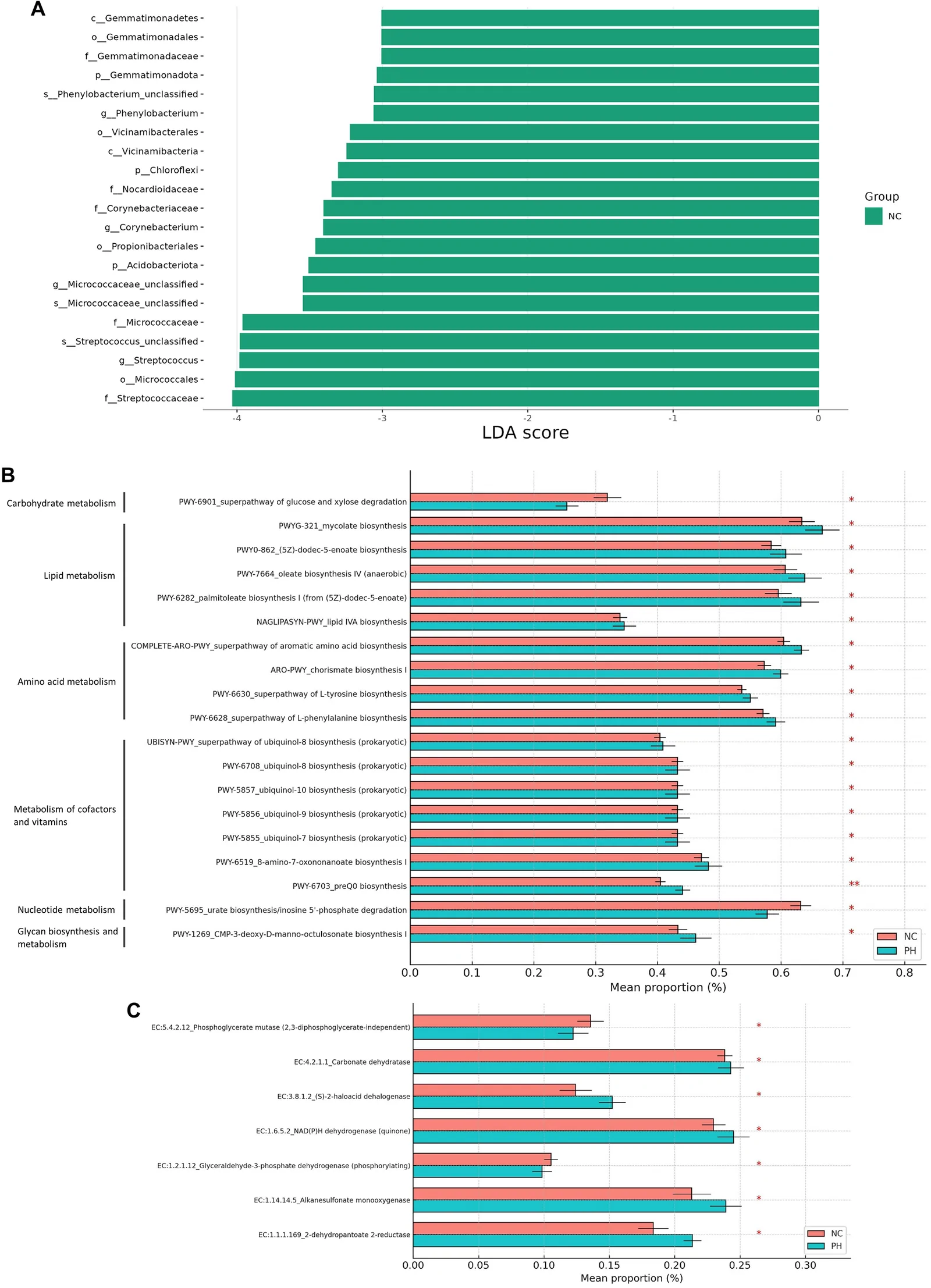
**(A)** Bar plots showing the linear discriminant analysis (LDA) scores of taxa that differed significantly between the two groups and achieved an LDA score greater than 3. **(B, C)** Differences in the predicted abundances of KEGG pathways and EC categories between the two groups. Statistical significance is indicated by asterisks, where * indicates *P* < 0.05 and ** indicates *P* < 0.01.

LEfSe is an analytical approach that is designed to identify and interpret potential biomarkers in high-dimensional datasets. This method integrates statistical testing for differential abundance with an effect-size estimation that reflects the contribution of each discriminative taxon to group separation, thereby highlighting taxa that are not only statistically significant but also biologically meaningful. In the PH group and the NC group, three phyla and four discriminative taxa at the genus or family level achieved an LDA score greater than 2.0, and the NC group was characterized by higher abundances of Gemmatimonadota, Chloroflexi, and Acidobacteriota, as well as enriched *Streptococcus*, Micrococcaceae, *Phenylobacterium*, and *Corynebacterium*, whereas no PH-specific taxa were identified (**Figure 3A**).

We further performed species correlation analyses across all detected microorganisms, and the genus-level co-occurrence network analysis showed that *Lactococcus* was significantly and negatively correlated with multiple opportunistic pathogens or environmental-associated genera (**Figure 2D**). *Lactococcus* displayed inverse association patterns with several genera, including *Burkholderia*–*Caballeronia*–*Paraburkholderia* (*r* = −0.912, *P* < 0.0001), *Stenotrophomonas* (*r* = −0.891, *P* < 0.0001), *Mycobacterium* (*r* = −0.886, *P* < 0.0001), *Methylobacterium*–*Methylorubrum* (*r* = −0.837, *P* < 0.0001), *Pseudomonas* (*r* = −0.795, *P* < 0.001), *Novosphingobium* (*r* = −0.681, *P* < 0.001), and *Myroides* (*r* = −0.648, *P* < 0.001), indicating that, at the individual level, an increased abundance of *Lactococcus* often coincided with decreased relative abundances of other taxa. This pattern may reflect two mutually exclusive community states in which a *Lactococcus*-dominant, lactic acid bacteria-enriched configuration contrasts with a community dominated by Gram-negative or environmental microorganisms. Given that lactic acid bacteria can produce lactate, modulate mucosal immunity, and strengthen the intestinal barrier, these negative associations partially support the ecological hypothesis that *Lactococcus* may serve as a potentially protective microbial signature in the early-life neonatal gut microbiota (Kirtzalidou et al., 2011; Alcon-Giner et al., 2020). This hypothesis still requires further validation.

### 3.4 Microbial functional profiling of the early-life neonatal gut microbiota in neonates born to CHD-PAH mothers

Predicted functional profiling of first-pass meconium indicated that the early-life neonatal gut microbiota of neonates born to mothers with pulmonary hypertension (PH) differed systematically from that of neonates born to mothers with congenital heart disease alone (NC) across multiple core metabolic pathways. Specifically, the PH group showed significant enrichment of several ubiquinone biosynthesis pathways, which suggests enhanced capacity for electron transport and redox regulation within the microbial community. The *de novo* biosynthesis of aromatic amino acids and their precursor chorismate was also consistently elevated in the PH group, which may indicate a potential upregulation of protein synthesis and the supply of precursors for diverse signaling molecules. In addition, the PH group exhibited broadly increased biosynthesis of unsaturated fatty acids, mycolate, and lipopolysaccharide-related structures such as lipid A and KDO, which collectively implies strengthened microbial capacity for membrane lipid assembly and endotoxin structural construction. In contrast, the NC group was relatively enriched in catabolic pathways involving glucose/xylose degradation and urate/IMP degradation, which reflects a metabolic profile that is more oriented toward substrate utilization and nitrogen-related metabolism (**Figure 3B**).

Based on the predicted functional analysis, we compared the relative abundances of several key metabolic enzymes in the meconium microbiota between the NC group and the PH group. The results showed that 2-dehydropantoate 2-reductase (EC:1.1.1.169), which participates in pantothenate/coenzyme A biosynthesis, was slightly higher in the PH group than in the NC group, and that alkanesulfonate monooxygenase (EC:1.14.14.5), which is related to organosulfonate degradation and sulfur metabolism, also exhibited an increasing trend in the PH group. Two key glycolytic enzymes, glyceraldehyde-3-phosphate dehydrogenase (EC:1.2.1.12) and phosphoglycerate mutase (EC:5.4.2.12), were highly represented in both groups, and the predicted abundance of GAPDH was modestly higher in the PH group. The respiratory chain-associated NAD(P)H dehydrogenase (quinone) (EC:1.6.5.2) was markedly enriched in the PH group relative to the NC group, which indicates that the PH-associated microbial community may possess enhanced redox homeostasis and energy metabolism. In addition, the relative abundances of (S)-2-haloacid dehalogenase (EC:3.8.1.2), which is implicated in the detoxification of halogenated organic acids, and carbonate dehydratase (EC:4.2.1.1), which catalyzes the interconversion of CO_2_ and HCO_3_^-^, were generally higher in the PH group (**Figure 3C**). Collectively, these enzymatic profiles suggest that the early-life neonatal gut microbiota in the PH group shows a tendency toward augmented capacities in carbohydrate metabolism, energy production, coenzyme biosynthesis, and xenobiotic degradation.

## 4 Discussion

In this study, we characterized the early-life neonatal gut microbiota in offspring of pregnancies complicated by congenital heart disease-associated pulmonary arterial hypertension and compared it with that of offspring born to mothers with congenital heart disease alone. The main findings were that the PH group showed lower alpha diversity, no significant between-group difference in overall beta diversity, markedly lower *Streptococcus* abundance, and potential differences in predicted functional profiles. These results extend current evidence on the influence of adverse maternal cardiovascular conditions on early-life microbial colonization and provide a basis for further mechanistic investigation.

All 40 meconium samples in our cohort showed detectable colonization, which supports the possibility that neonatal meconium may contain bacterial DNA before birth (Collado et al., 2016; Turunen et al., 2021). Compared with the NC group, the PH group exhibited lower alpha diversity, as reflected by decreases in the Shannon index and Observed species; although richness-oriented metrics such as Simpson did not show significant between-group differences, the marked reduction in the Shannon index suggests a concomitant decline in diversity and evenness, which are characteristic features of gut microbial dysbiosis. The two groups did not show an obvious difference in beta diversity. At the community level, Bray–Curtis PCoA showed substantial overlap between the two groups, and PERMANOVA did not indicate a statistically significant difference (R^2^ = 0.0145, P = 0.66). Dispersion analysis likewise showed no significant between-group difference in within-group heterogeneity (NC = 0.572, PH = 0.532; *P* = 0.201).

Although the overall community structures of the PH and NC groups appeared to be slightly different, these differences did not reach statistical significance. The dominant phyla identified in meconium included Firmicutes, Actinobacteriota, Proteobacteria, and Bacteroidota, and Proteobacteria was the most abundant among them; at the genus level, we detected *Enterobacter*, *Staphylococcus*, *Bifidobacterium*, and *Mycobacterium*, which are common taxa in neonatal gut ecosystems and are consistent with previous studies (Shi et al., 2018; Tapiainen et al., 2018). Overall, the early-life neonatal gut microbiota in the PH and NC groups shared broadly similar community architectures, and no statistically significant differences were observed in the relative abundances of dominant taxa. At the genus level, *Pantoea* was the most predominant taxon, and a *Pantoea*-dominated community is not typically considered a common maternal-derived component, which suggests that this genus may be introduced from environmental sources. *Pantoea* comprises a diverse group of yellow-pigmented, rod-shaped Gram-negative bacteria within the Enterobacteriaceae family (Walterson and Stavrinides, 2015), and it is widely distributed in plants, soil, and the human gastrointestinal tract. Because *Pantoea* can also be isolated from hospital environments, it has been implicated in opportunistic infections, and clinical case reports have documented that this genus can cause opportunistic bloodstream infections in neonatal intensive care units and may also be associated with allergic manifestations and hypersensitivity pneumonitis (Dutkiewicz et al., 2016; Mani and Nair, 2021). However, since the relative abundance of *Pantoea* did not differ significantly between the PH group and the NC group, its enrichment in our cohort may be more plausibly attributed to shared environmental exposures rather than disease-specific effects.

In contrast, the relative abundance of *Streptococcus* in neonatal meconium was significantly lower in the PH group than in the NC group. *Streptococcus* is a common pioneer colonizer in early neonatal life that is thought to originate primarily from maternal vaginal, oral, and skin microbiota (Turunen et al., 2021), although it is also recognized as a frequent opportunistic genus in the gut. Pregnant women with pulmonary arterial hypertension tend to have higher rates of preterm birth and cesarean delivery, which may weaken the vertical transmission of *Streptococcus* to the neonate and thereby partly explain the reduced abundance observed in the PH group (Wang et al., 2020; Greenfield et al., 2024). On the other hand, previous studies have shown that certain *Streptococcus* strains can enhance mucosal immunity by downregulating pro-inflammatory cytokines such as TNF-α and IL-6, by promoting intestinal mucosal IgA production, and by improving the integrity and function of the gut barrier (Li et al., 2022; Mazziotta et al., 2023). In the present study, its relative abundance was markedly lower in the PH group, suggesting that pregnancies complicated by CHD-associated pulmonary hypertension may be associated with an altered early colonization pattern. These findings may suggest that neonates born to mothers with pulmonary arterial hypertension could have weakened early intestinal barrier defenses against pathogens. Given that some *Streptococcus* species have been linked to mucosal immune modulation and intestinal barrier homeostasis, this finding may have potential biological relevance, although mechanistic validation is still required. In addition, several low-abundance environmental-associated microorganisms differed significantly between groups, which may indicate that mothers with pulmonary arterial hypertension are more likely to experience more severe hypoxemia and to receive pharmacologic treatment and inpatient medical interventions, and these factors may reshape the placental milieu and perinatal exposures in ways that reduce opportunities for colonization by certain environmental taxa in meconium. The clinical implications of these observations remain uncertain and require validation in larger cohorts and studies that apply higher-throughput approaches.

In this study, the predicted abundances of several core metabolic enzymes in the meconium microbiota of the PH group were overall higher than those in the NC group, including key glycolytic enzymes, respiratory chain NAD(P)H dehydrogenases, and enzymes involved in pantothenate and coenzyme A biosynthesis, which suggests that the early-life neonatal gut microbiota in the PH group may possess a more active potential for carbohydrate catabolism and energy metabolism. Enzymes related to sulfur metabolism and the degradation of halogenated organic compounds were also relatively enriched in the PH group, and the predicted abundance of carbonic anhydrase was increased. Because gut microorganisms have been reported to harbor genes and biochemical capacities that mediate the transformation of halogenated substrates (Wolf et al., 2022; Wolfson et al., 2022), and because carbonic anhydrase is a conserved functional enzyme that participates in the interconversion of CO_2_ and HCO_3_^-^ and contributes to pH regulation (Supuran and Capasso, 2017), these functional shifts may reflect chronic intrauterine stressors in pregnancies complicated by PAH, such as sustained hypoxia and acid–base fluctuations, which could bias early microbial colonization toward functional lineages that are better equipped to utilize non-canonical substrates and to maintain redox and pH homeostasis. However, because PICRUSt2 infers function from 16S rRNA gene data rather than directly measuring gene content, expression, or metabolites, these findings should be interpreted cautiously and regarded as hypothesis-generating. Future studies integrating metagenomic sequencing, metatranscriptomics, and metabolomics will be important for direct validation.

This study incorporated a high-risk clinical population with congenital heart disease-associated pulmonary arterial hypertension, for which evidence in the field of maternal–fetal microecology remains relatively scarce, and it leveraged first-pass neonatal meconium as a temporal “window” that reflects the earliest colonization status from the intrauterine to the perinatal period, thereby addressing a critical knowledge gap regarding the impact of PAH on the early-life neonatal gut microbiota and providing a new microbiome-based line of evidence that may help explain the potential biological mechanisms underlying adverse maternal–fetal outcomes in CHD-PAH.

Several limitations should be acknowledged. Several limitations should be acknowledged. First, because group assignment for pulmonary arterial hypertension primarily relied on echocardiography-estimated pulmonary arterial pressure rather than right heart catheterization, measurement deviation and error may have resulted in misclassification of some cases, which could have attenuated the true effect size or introduced bias. Second, although the overall baseline characteristics were generally comparable between groups, gestational age at delivery as well as neonatal length and birth weight differed significantly between the PH and NC groups, and these differences were not adjusted for in the present analysis because of the relatively small sample size and the exploratory nature of the study. Therefore, a potential confounding effect on the microbiome findings cannot be excluded, since fetal maturity and neonatal growth status may influence early microbial colonization. In addition, although moderate-to-severe PAH is often accompanied by hypoxemia and metabolic disturbances in clinical settings, not all cases in our PH group exhibited hypoxemia or overt metabolic dysregulation. This suggests that heterogeneity in disease severity and systemic metabolic status persisted within the group and may have influenced microbial features. Future studies integrating metabolomics and metagenomic sequencing may provide important complementary insights. Metabolomics could help characterize microbiota-related metabolic outputs and host–microbiota interactions, whereas metagenomic sequencing could offer higher-resolution taxonomic and functional information beyond 16S rRNA-based inference. The integration of these approaches may help clarify the biological significance and mechanistic basis of the microbiome alterations observed in the present study.

## 5 Conclusion

This 16S rRNA sequencing study suggests that congenital heart disease-associated pulmonary arterial hypertension may influence the establishment of the early-life neonatal gut microbiota. Neonates in the PH group exhibited reduced microbial diversity in meconium and limited compositional differences at the genus level. Although the overall phylum-level architecture was broadly similar between groups, the relative abundance of *Streptococcus* was significantly lower in the PH group. Predicted functional profiling suggested potential differences in dominant metabolic pathways. Taken together, these findings provide new microbiome-based evidence and directions for elucidating the biological mechanisms that may underlie adverse maternal–fetal outcomes associated with CHD-PAH.

## Data Availability

The raw data supporting the conclusions of this article will be made available by the authors, without undue reservation.
