# Correction: Altered early-life gut microbiota in offspring of pregnancies complicated by CHD-associated pulmonary hypertension

**DOI:** 10.3389/frmbi.2026.1932978

**Published:** 2026-07-27

**Authors:** Yiyang Han, Haofeng Zhang, Jun Zhang

**Affiliations:** 1 Department of Cardiac Surgery, Beijing Anzhen Hospital, Capital Medical University, Beijing, China; 2 Beijing Institute of Heart, Lung and Blood Vessel Diseases, Beijing, China

**Keywords:** 16S rRNA, congenital heart disease, early-life neonatal gut microbiota, pregnancy, pulmonary arterial hypertension

In the **Funding** section, the funding number was omitted for the Capital’s Funds for Health Improvement and Research. The correct number is “CFH2024-2-2068”. The correct **Funding** statement reads:

“The author(s) declared that financial support was received for this work and/or its publication. This study was supported by the Capital’s Funds for Health Improvement and Research (CFH2024-2-2068).”

The original version of this article has been updated.

