# Supplementary figures and images for: Altered early-life gut microbiota in offspring of pregnancies complicated by CHD-associated pulmonary hypertension
Source: Front Microbiomes. 2026 May 29;5:1785707. doi: 10.3389/frmbi.2026.1785707 (PMC13260587; doi:10.3389/frmbi.2026.1785707)

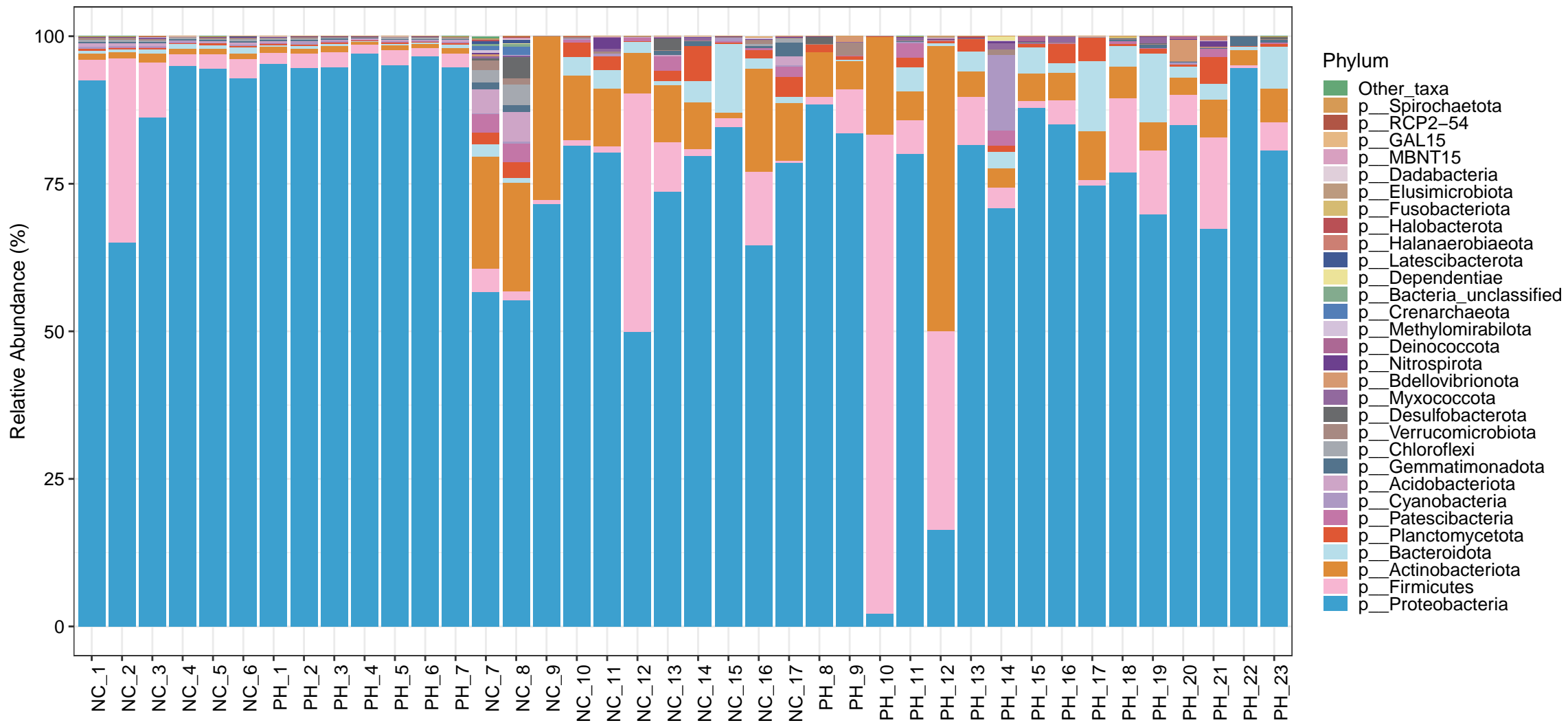

Supplement: Supplementary file 1 [file DataSheet1.pdf]

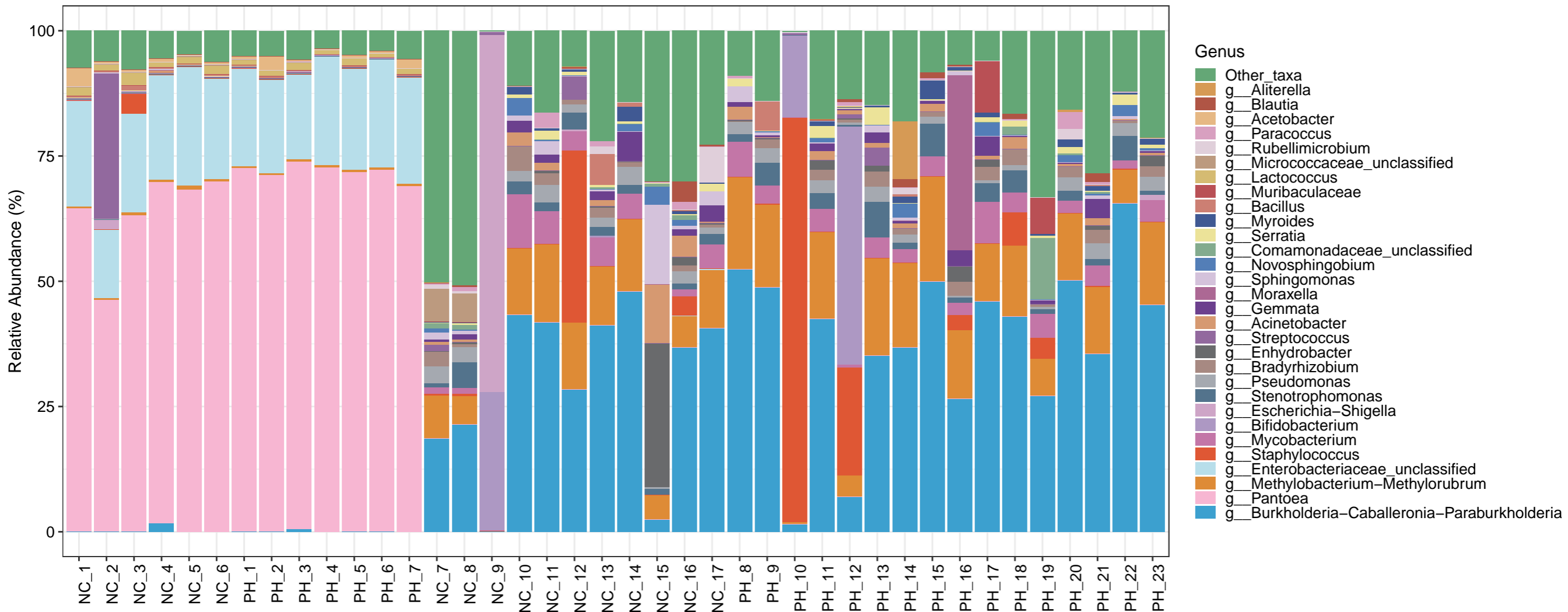

Supplement: Supplementary file 2 [file DataSheet2.pdf]
